# Common Variant rs9939609 in Gene FTO Confers Risk to Polycystic Ovary Syndrome

**DOI:** 10.1371/journal.pone.0066250

**Published:** 2013-07-01

**Authors:** Tao Li, Keliang Wu, Li You, Xiuye Xing, Peng Wang, Linlin Cui, Hongbin Liu, Yuqian Cui, Yuehong Bian, Yunna Ning, Han Zhao, Rong Tang, Zi-Jiang Chen

**Affiliations:** 1 Center for Reproductive Medicine, Provincial Hospital Affiliated to Shandong University, Jinan, China; 2 National Research Center for Assisted Reproductive Technology and Reproductive Genetics, Jinan, China; 3 The Key Laboratory for Reproductive Endocrinology of Ministry of Education, Jinan, China; 4 Shandong Provincial Key Laboratory of Reproductive Medicine, Jinan, China; 5 Department of Obstetrics and Gynecology, Renji Hospital, School of Medicine, Shanghai Jiaotong University, Shanghai, China; CAEBi, Spain

## Abstract

**Background:**

Fat mass and obesity-associated gene (*FTO*) has been associated with obesity, especially the common variant rs9939609. Polycystic ovary syndrome (PCOS) is a complex endocrine-metabolic disorder and over 50% of patients are overweight/obese. Thus *FTO* is a potential candidate gene for PCOS but their relationship is confusing and remains to be clarified in different population with a large sample size**.**

**Method:**

This study was performed adopting a two-stage design by genotyping SNP rs9939609. The first set comprise of 741 PCOS and 704 control subjects, with data from our previous GWAS. The second phase of replication study was performed among another independent group of 2858 PCOS and 2358 control subjects using TaqMan-MGB probe assay. All subjects are from Han Chinese**.**

**Results:**

The less meaningful association of *FTO* rs9939609 and PCOS discovered in GWAS (P = 2.47E-03), was further confirmed in the replication study (P = 1.86E-09). Using meta-analysis, the P-meta value has reached 6.89E-12, over-exceeding the genome-wide association level of 5.00E-8. By combination, the P value was 1.26E-11 and after BMI adjustment it remained significant(P = 1.82E-06). To further elucidate whether this association is resulted from obesity or PCOS per se, the samples were divided into two groups–obese and non-obese PCOS, and the results were still positive in obese group (P obese = 5.81E-05, OR = 1.55), as well as in non-obese PCOS group (P non-obese = 7.06E-04, OR = 1.28)**.**

**Conclusion:**

Variant rs9939609 in *FTO* is associated with PCOS in Chinese women, not only in obese PCOS subjects, but also in non-obese cases**.**

## Introduction

Polycystic ovary syndrome (PCOS) is a complex endocrine-metabolic disease affecting 6–8% women of reproductive age [1]. Characterized by clinical and/or biochemical androgen excess, ovulatory dysfunction and polycystic ovaries, PCOS patients is also associated with an increased risk of overweight/obesity, insulin resistance and type 2 diabetes mellitus (T2DM) [1], [2], [3], [4]. Over 50% PCOS cases are overweight/obese [5]. Evidence implies obesity, interacting with T2DM and endocrine disorders, is an important factor in the etiology of PCOS [6], [7]. And the prevention and treatment of obesity will benefit PCOS patients [8]. Family-based and case-control association studies suggest genetic factors contribute to both obesity and PCOS, which implicates a shared genetic predisposition in their concurrence [9], [10], [11].

Fat mass and obesity associated gene (*FTO*), located on chromosome 16q12.2, is expressed in a wide range of tissues including adipose tissue, muscle, pancreas, and liver, with the highest concentrations in the hypothalamus [12]. *FTO* has been wildly identified to be associated with body mass index (BMI) and obesity by large scale genome-wide association studies and a wealth of replication studies in several ethnicities, including Chinese [13], [14], [15], [16], [17], [18]. Besides obesity, *FTO* also confers risk to T2DM [19], [20], [21], although this association may be independent of BMI in East and South Asian [22]. In above studies common variant rs9939609 is representative SNP marker. Considering both obesity and T2DM are major complications of PCOS, *FTO* may also confer risk to PCOS.

The association of *FTO* and PCOS has been widely investigated, but the results remain controversial. Variant rs9939609 of *FTO* was proved to be associated with PCOS in U.K. women [23], while the association was not verified in American and European Caucasian [24], [25]. In Chinese this result has been replicated but became not significant after adjustment by BMI [26]. All of these studies have been characterized by a limited sample size and statistical power. Therefore, enlarged sample size is requested to get a credible interpretation.

The aim of this study was to investigate the relationship between *FTO* variant and PCOS whether on the effect of BMI. After reviewing our previous discovery cohort of GWAS (Table S1 in file S1) [27], we conducted a replication study. A total of 3599 PCOS cases and 3082 control subjects were enrolled. Obesity-related traits such as BMI, waist–hip ratio, glucose and lipid profiles in different genotypes of rs9939609 were also analyzed in PCOS patients.

## Materials and Methods

### Subjects

Based on the Rotterdam Consensus proposed in 2003 [28], diagnosis of PCOS were determined as two of the following characteristics existence at least, oligo−/aovulation (OA), polycystic ovarian morphology (PCO), clinical or biochemical hyperandrogenism (HA). The diagnosis of PCOS was made only when the other etiologies for hyperandrogenemia and ovulatory dysfunction were excluded, i.e. congenital adrenal hyperplasia, 21-hydroxylase deficiency, androgen-secreting tumors, Cushing’s syndrome, thyroid disease, and hyperprolactinemia. A total of 3599 women with PCOS, among which 741 were originated from our first GWAS and 2858 from the independent replication study, were recruited at the reproductive medical center of Shandong Provincial Hospital affiliated to Shandong University from Jun 2009 to May 2012.

A total of 3082 age-matched healthy women, comprising of our first GWAS (704) and the replication study (2378), were enrolled of the same time period. All subjects in control group had regular menstrual cycle (26–35 days) and normal ovarian morphology. Total testosterone and modified Ferriman-Gallwey score were evaluated for exclusion of hyperandrogenism.

All of participants were unrelated individuals of reproductive age with no hormonal therapy for at least three months prior to the test. Medication history and history of weight loss for all subjects were collected to exclude these who have had weight or glucose affecting measures such as those taking metformin or any other oral hypoglycemic agents.

### Ethics Statement

The study was approved by the Institutional Review Board of Reproductive Medicine of Shandong University. All participants were informed of the genetic detection and written informed consent was obtained.

### Clinical and Biochemical Measurements

Height and weight were measured with participants dressed in underwear without shoes. BMI (kg/m^2^) was calculated as the weight in kilograms divided by the square of height in meters. Obesity was defined as BMI≥25 kg/m^2^ according to 1997 WHO criteria for Chinese [29]. Waist circumference was measured midway between the lowest rib and the iliac crest. Hip circumference was the longest measurement of hip. Waist-hip ratio (WHR) was defied as waist circumference divided by hip circumference.

Peripheral blood samples were collected during day 2–4 of spontaneous cycles or after withdrawal of bleeding from all subjects after a 12-h overnight fast. Follicle stimulating hormone (FSH), luteinizing hormone (LH), prolactin, testosterone (T) and estradiol (E2) of all subjects were measured by a chemi-luminescent analyzer (Beckman Access Health Company, Chaska, MN, USA).

### Glucose Metabolic and Lipid Measurements

The glucose metabolic and lipid indices were measured for woman with PCOS. Blood glucose and insulin of fasting and 2 hours after a 75-g oral glucose tolerance test (OGTT) was measured (AU640 automatic biochemistry analyzer; Olympus Company, Hamburg, Germany.). Insulin resistance was assessed using the homeostasis model (HOMA-IR = fasting blood glucose (FBG mmol/L)×Fasting insulin (FINS mIU/L)/22.5). Serum cholesterol (CHOL), triglycerides (TG), low density lipoprotein (LDL) and high density lipoprotein (HDL) were evaluated by enzymatic method. Of the controls whose medical conditions were normal by body examination in our department, we only extracted DNA for genotyping.

### SNP Genotyping

Genomic DNA was extracted with QIAamp DNA mini kit (QIAGEN, Hilden, Germany) according to the manufacturer’s protocol. Subjects of GWAS were genotyped by Affymetrix Genome-Wide Human SNP Array 6.0 [27]. For the replication study, variant rs9939609 was analyzed by TaqMan-MGB probe assay (Invitrogen trading, Shanghai) by available primers and probes. The reactions were performed on 384-well plates, Roche Lightcycle 480 carried out by pre-incubation at 95°C for 4 min followed by 45 cycles of denaturation at 95°C for 15 s, annealing, extension and detection for 40 s at 60°C. 100 PCOS and 100 control participants from the GWAS cohort were re-tested to make sure the coordinance of different methods. Direct sequencing of 5% randomly selected samples were applied to validate the genotyping assays. And the success rate was 99.5%.

### Statistical Analysis

Continuous parameters of patients and controls are expressed as means±SD. Genetic Power Calculator [30] was applied for the sample size estimation and case-control genetic power calculation. PLINK (v.1.07, http://pngu.mgh.harvard.edu/purcell/plink) was applied to calculate the Hardy-Weinberg equilibrium (P), allele frequency differences and genotype differences. Odd ratios (ORs) with 95% CIs (confidence intervals) are presented. Meta-analysis for the all samples was performed by the fixed effects Cochran-Mantel-Haenszel (CMH) test by PLINK. Genetic models were divided into additive (+/+ vs. +/− vs. −/−), dominant (+/+ plus +/− vs. −/−) and recessive (+/+ vs. +/− plus −/−). By the additive effects of allele dosage, the logistic regression for disease trait was conducted to exclude the potential confounding effects of age and/or body mass index. The genotype models were applied for the phenotype analysis which was compared by one-way ANOVA or student T test, SPSS, 16.0 (SPSS Inc., Chicago, IL, USA). And P<0.05 was regarded as statistically different.

## Results

### Basic Clinical Measurements

3599 PCOS patients and 3082 controls were recruited and the case-control genetic power has reached 0.8 at P = 1E-5 level. Basic clinical measurements were compared and presented in Table 1.

**Table 1:** Basic clinical characteristics comparisons between PCOS and Control subjects.

Characteristics	Groups(Mean±SD)		P	P adj
	PCOS(3599)	CONTROL(3082)		
AGE (years)	28.35±3.75	31.33±4.69	<0.001	–
BMI (kg/m^2^)	24.81±4.29	22.73±3.15	<0.001	–
WAIST (cm)	84.14±11.10	77.80±7.69	<0.001	0.13
WHR	0.87±0.06	0.84±0.05	<0.001	0.9
FSH (IU/L)	6.30±1.90	7.30±3.02	<0.001	<0.001
LH (IU/L)	10.38±5.81	4.89±2.37	<0.001	<0.001
T (ng/dl)	55.51±24.83	21.53±12.66	<0.001	<0.001

SD = standard error. WHR = wasit-hip ratio. FSH = follicle-stimulating hormone. LH = luteinizing hormone. T = testosterone. P adj, adjusted P value by age and BMI in logistic regression.

In PCOS group, 44% (1577/3587) subjects were obese (BMI≥25 kg/m^2^) while the percentage in control was 21.4% (660/3082). PCOS women had significantly higher levels of luteinizing hormone and testosterone. In logistic regression, the differences of waist circumference and WHR were not significant after age and BMI adjustment (P _wasit = _0.13, P _WHR_ = 0.9), which implies BMI may well stand for the central obesity indices in our study subjects.

### Allele and Genotypes

Data of our study were consisted of two parts that were GWAS data (741 PCOS cases vs. 704 control subjects) and replication study (2858 PCOS case vs. 2378 control subjects). The observed genotype distribution was in consistent with Hardy–Weinberg equilibrium in both GWAS and replication study respectively. Minor allele frequency (MAF) comparison was presented in Table 2. In the two parts, the association of rs9939609 and PCOS has been discovered (P _GWAS_ = 2.47E-03, OR = 1.43; P _replication_ = 1.86E-09, OR = 1.44). The P value of meta-analysis is 6.89E-12 (I^2^ = 92%), which has reached the GWAS standard of significance (P<5.0E-8).

**Table 2:** Allele comparison of rs9939609 in GWAS and replication study.

	Risk Allele	MAF PCOS	MAF control	P	ORs 95% CI	P adj	P meta
GWAS	A	0.131	0.095	**2.47E-03**	1.43	**2.30E-02**	**6.89E-12**
					1.13–1.81		
Replication study	A	0.141	0.102	**1.86E-09**	1.44	**4.00E-04**	
					1.28–1.68		

GWAS = genome-wide association study. In GWAS study, PCOS: control = 741∶704. In replication study, PCOS: control = 2858∶2378. MAF = minor allele frequency. ORs = Odds Ratios. 95% CI = confidence interval. P adj, adjusted P value by BMI and age in logistic regression. P meta, meta-analysis of GWAS and replication study by PLINK.

In order to eliminate the function of BMI in the association, the two cohorts were combined and analyzed (Table 3). After adjustment of BMI, the difference between PCOS and control was still significant (P adjusted = 1.82E-06). The OR of combined study was similar to the above (P combined = 1.26E-11, OR = 1.44).

**Table 3:** Allele and genotype analysis of combined data.

SNP	Comparisons	PCOS	control	P	OR	Adjustment study	
		Minor/Major	Minor/Major		95%CI	STAT	P
rs9939609 A/T	ALLELE	1001/6197	621/5543	**1.26E-11**	1.44	4.77	**1.82E-06**
					1.30–1.60		
	ADD	67/867/2665	29/563/2490	**1.09E-10**	–	–	–
	DOM	934/2665	592/2490	**5.95E-11**	–	–	–
	REC	67/3532	29/3053	**1.62E-3**	–	–	–

Allele, the data was presented as A/T in the two groups. ADD, the data was presented by the additive genotype model (AA/AT/TT) in the three groups. DOM, the data was presented by the dominant genotype model (AA+AT/TT) in the two groups. REC, the data was presented by the recessive genotype model (AA/AT+TT) in the two groups. Adjustment study, adjusted by BMI in logistic regression.

To further illuminate the influence of BMI, the PCOS subjects were divided into obese group and non-obese groups. Compared with BMI matched controls, in obese PCOS group, MAF was higher than BMI matched controls (P _obese_ = 5.81E-05, OR = 1.55; Table 4), and the difference was still dominant after age and BMI adjustment (P _adjusted_ = 4.43E-05).

**Table 4:** Allele comparisons of PCOS and control divided into obese and non-obese group.

	MAF		P	OR	P adj
	PCOS	control		95% CI	
Obese group	0.163	0.114	**5.81E-05**	1.55 (1.25–1.92)	**4.43E-05**
Non-obesegroup	0.121	0.097	**7.06E-04**	1.28 (1.11–1.48)	**2.74E-04**

In obese group, PCOS: control = 1577∶660. In non-obese group, PCOS: control = 2010∶2422. P adj, adjusted P value by BMI and age in logistic regression. MAF = minor allele frequency. OR = odds risk. 95% CI = confidence interval.

Similarly in the non-obese PCOS, the the P_non-obese_ value was 7.06E-04 (OR = 1.28). And the difference in non-obese subjects was even more evident after BMI and age adjustment (non-obese group, P_adjusted_ = 2.74E-04).

### Clinical and Metabolic Measurements in PCOS

There was a gradient in weight and BMI with the highest in homozygous A allele individuals and the lowest in individuals with homozygous T allele in PCOS (1.53 kg increase in weight, P = 3.6E-05; 0.76 kg/m2 increase in BMI, P = 1.7E-04). Apparently, BMI and waist circumstance increased along with increase of the risk allele A (Table S2 in file S1). However, there were no other differences exiting in the parameters of endocrine and metabolic characteristics including glucose metabolic indices and lipid profiles (Table S3 in file S1).

## Discussion

Polycystic ovary syndrome, as well as individual susceptibility to obesity, is thought to be caused by interactions between genetic make-up and the environment. Recently, GWASs on PCOS have identified a series of susceptibility loci, whereas few are obesity related [27], [31]. If PCOS and obesity share a common genetic background, replicating the obesity susceptibility genes in PCOS patients is a helpful method to discover risk variants, which needs well exclusion of confounding factors. *FTO* is a common candidate gene for obesity and BMI [17]and probably contribute to PCOS. In this study, we recruited a large number of samples and identified that *FTO* is the susceptibility gene for PCOS regardless of the presence of obesity.

In our study, with a cohort of total 3599 PCOS cases and 3083 controls, we identified strong evidence that risk allele A of rs9939609 confers higher risk to PCOS (P_combined_ = 1.26E-11, ORs = 1.44; P_meta_ = 6.89E-12), even after BMI adjustment (P = 1.82E-6). SNP rs9939609 has been the common variant in the identification of *FTO* as the susceptibility gene to obesity and T2DM [13], [14], [15], [16], [17], [18], [19], [20], [22]. And the genotype of rs9939609 would influence *FTO* expression in transcript level [32], [33] and thus is related to fat cell lipolysis [34]. Studies also proved that rs9939609 related to increased food responsiveness and better emotional control [35]. As the association has been identified in our study, functional studies about rs9939609 in PCOS would help to the explain the etiology.

The relationship of *FTO* and PCOS has been investigated, but the association has been influenced by BMI to a large extent. As concluded in Table 5, the association of *FTO* with PCOS susceptibility has not been observed in Caucasian and Korean women [24], [25], [36], [37]. Even the association has been discovered in studies of Barber et al. and Yan et al., it became nonsense in obese PCOS by BMI stratification analysis, indicating that the predominant of *FTO* on PCOS susceptibility mainly mediated through BMI [23], [26]. As *FTO* has been identified to be susceptibility gene to obesity, the entanglement of obesity and PCOS suggests that adjustment analysis by BMI would not be as effective. It is necessary to stratify all participants by obesity to eliminate the bias due to the genetic effect of the rs9939609 SNP with obesity. In our study the strong association was some extent impacted by BMI, but still evident in non-obese PCOS (P = 2.74E-04), as well as in obese group (P = 4.43E-05). As rs9939609 has been the representative variant of *FTO*, a direct effect of *FTO* conferred to PCOS has been revealed regardless of obesity or BMI.

**Table 5:** Previous association studies of *FTO* and PCOS.

	SNP	Population	Sample size	MAF(%)		P	Adjustment study	BMI-stratified study	
			PCOS/CTRL	PCOS	CTRL		P	High-BMI	Low-BMI
Barber et al. (22)	rs9939609	Caucasian	463/1336	46.3	40.0	**7.4E-04**	**2.9E-03**	**2.9E-04**	0.11
Attaoua et al. (31)	rs1421085	Caucasian	207/100	49	46	>0.05	>0.05	**<0.04**	>0.05
Yan et al (25).	rs9939609	Chinese	215/227	15.1	9.9	**0.019**	0.286	**0.016**	0.065
Saxena et al. (24)	rs9939609	Caucasian and African	510/448	43.1	39.6	0.811	1	–	–
Ewens et al.(23)	rs9939609	Caucasian	439 trios395/176	45.6; –	-	0.575; –	–	–	–
Kim et al. (32)	rs1421085	Korean	377/386	15.7	15.2	0.789	–	–	–
Current study	rs9939609	Chinese	3599/3082	13.9	10.1	**1.26E-11**	**1.82E-06**	**5.81E-05**	**7.06E-04**

CTRL = control; MAF = minor allele frequency.

*FTO*, as confirmed by our study, confers risk to the pathogenesis of PCOS per se; at the meanwhile, increased BMI may have synergetic effect on PCOS by *FTO* function. Obesity is associated with hyperandrogenism and menstrual disturbance of PCOS and worsens PCOS complications such as T2DM [4], [7], [22], we hypothesize that *FTO* should be one of the molecular determinants linking obesity to PCOS. *FTO* is associated with eating habit and is important to the control of energy homeostasis [38]. It acts as a transcriptional coactivator in epigenetic regulatory processes [39] and nucleic acid repair or modification processes [40], which is important in modulating the hyperandrogenism and involves in the ovarian dysfunction of PCOS [41]. The role of *FTO* in general mechanism and epigenetic regulation suggests that *FTO* may be a pleiotropic factor involved in various diseases such as PCOS, obesity and T2DM. However, the function and biological pathways of *FTO* protein in PCOS has not been fully considered. Further studies on *FTO* in PCOS patients will provide possible interpretation for its etiology and interaction of obesity and PCOS.

Clinical and metabolic measurements have been analyzed for the understanding of *FTO* effects in PCOS. Different from the study of Yan et al [26], our study confirmed the genotype-dependent relationship between rs9939609 risk allele and BMI of PCOS, which has also been revealed in the studies and meta-analysis of the Caucasian cases [23], [42], [43]. Interestingly, it was not found in control group yet. The increases of BMI and weight, similar to Tan’s report [42], were not as high as the meta-analysis of eight distinct cohorts by Wojciechowski et al. [43]. Considering the meta-analysis did not cover Asian patients, it may due to the different genetic backgrounds between ethnicities. Although Wehr et al. [44] demonstrated an increased hyperandrogenemia indices in PCOS group along with rs9939609, it was not shown in our study. The reason may due to the difference that hyperandrogenism is less prevalent in Chinese Han than Caucasians. As to other metabolic profiles, no significance was found in our study, nor other previous studies [45]. However, subjects enrolled in the study are reproductive-aged young people and few of them suffered from metabolic disorders. More profound researches are expected to learn the pathophysiological effect of *FTO* in PCOS and tracking PCOS women would help to clarify the relationship of *FTO* and metabolic disturbances.

In summary, we have demonstrated that variant rs9939609 in *FTO* is associated with PCOS in Chinese women. The association has been disclosed both in obese group and non-obese group. Nonetheless, more in-depth studies are required to elucidate the biological role of *FTO* playing on the interaction of obesity and PCOS.

## Supporting Information

File S1. (DOC)

## References

[1] Azziz R, Woods KS, Reyna R, Key TJ, Knochenhauer ES, et al (2004) The prevalence and features of the polycystic ovary syndrome in an unselected population. J Clin Endocrinol Metab 89: 2745–2749. 15181052 10.1210/jc.2003-032046

[2] Balen AH, Conway GS, Kaltsas G, Techatrasak K, Manning PJ, et al (1995) Polycystic ovary syndrome: the spectrum of the disorder in 1741 patients. Hum Reprod 10: 2107–2111. 8567849 10.1093/oxfordjournals.humrep.a136243

[3] Carmina E, Azziz R (2006) Diagnosis, phenotype, and prevalence of polycystic ovary syndrome. Fertil Steril 86 Suppl 1S7–8. 16798288 10.1016/j.fertnstert.2006.03.012

[4] Lim SS, Davies MJ, Norman RJ, Moran LJ (2012) Overweight, obesity and central obesity in women with polycystic ovary syndrome: a systematic review and meta-analysis. Hum Reprod Update 18: 618–637. 22767467 10.1093/humupd/dms030

[5] Shi Y, Guo M, Yan J, Sun W, Zhang X, et al (2007) Analysis of clinical characteristics in large-scale Chinese women with polycystic ovary syndrome. Neuro Endocrinol Lett 28: 807–810. 18063948

[6] Azziz R, Sanchez LA, Knochenhauer ES, Moran C, Lazenby J, et al (2004) Androgen excess in women: experience with over 1000 consecutive patients. J Clin Endocrinol Metab 89: 453–462. 14764747 10.1210/jc.2003-031122

[7] Barber TM, McCarthy MI, Wass JA, Franks S (2006) Obesity and polycystic ovary syndrome. Clin Endocrinol (Oxf) 65: 137–145. 16886951 10.1111/j.1365-2265.2006.02587.x

[8] Lim SS, Norman RJ, Davies MJ, Moran LJ (2013) The effect of obesity on polycystic ovary syndrome: a systematic review and meta-analysis. Obes Rev 14: 95–109. 23114091 10.1111/j.1467-789X.2012.01053.x

[9] Amato P, Simpson JL (2004) The genetics of polycystic ovary syndrome. Best Pract Res Clin Obstet Gynaecol 18: 707–718. 15380142 10.1016/j.bpobgyn.2004.05.002

[10] Vink JM, Sadrzadeh S, Lambalk CB, Boomsma DI (2006) Heritability of polycystic ovary syndrome in a Dutch twin-family study. J Clin Endocrinol Metab 91: 2100–2104. 16219714 10.1210/jc.2005-1494

[11] Walley AJ, Blakemore AI, Froguel P (2006) Genetics of obesity and the prediction of risk for health. Hum Mol Genet 15 Spec No 2: R124–130. 10.1093/hmg/ddl215 16987875

[12] Stratigopoulos G, Padilla SL, LeDuc CA, Watson E, Hattersley AT, et al (2008) Regulation of Fto/Ftm gene expression in mice and humans. Am J Physiol Regul Integr Comp Physiol 294: R1185–1196. 18256137 10.1152/ajpregu.00839.2007 PMC2808712

[13] Chang YC, Liu PH, Lee WJ, Chang TJ, Jiang YD, et al (2008) Common variation in the fat mass and obesity-associated (FTO) gene confers risk of obesity and modulates BMI in the Chinese population. Diabetes 57: 2245–2252. 18487448 10.2337/db08-0377 PMC2494679

[14] Peng S, Zhu Y, Xu F, Ren X, Li X, et al (2011) FTO gene polymorphisms and obesity risk: a meta-analysis. BMC Med 9: 71. 21651756 10.1186/1741-7015-9-71 PMC3118373

[15] Dina C, Meyre D, Gallina S, Durand E, Korner A, et al (2007) Variation in FTO contributes to childhood obesity and severe adult obesity. Nat Genet 39: 724–726. 17496892 10.1038/ng2048

[16] Scuteri A, Sanna S, Chen WM, Uda M, Albai G, et al (2007) Genome-wide association scan shows genetic variants in the FTO gene are associated with obesity-related traits. PLoS Genet 3: e115. 17658951 10.1371/journal.pgen.0030115 PMC1934391

[17] Walley AJ, Asher JE, Froguel P (2009) The genetic contribution to non-syndromic human obesity. Nat Rev Genet 10: 431–442. 19506576 10.1038/nrg2594

[18] Wen W, Cho YS, Zheng W, Dorajoo R, Kato N, et al (2012) Meta-analysis identifies common variants associated with body mass index in east Asians. Nat Genet 44: 307–311. 22344219 10.1038/ng.1087 PMC3288728

[19] Frayling TM, Timpson NJ, Weedon MN, Zeggini E, Freathy RM, et al (2007) A common variant in the FTO gene is associated with body mass index and predisposes to childhood and adult obesity. Science 316: 889–894. 17434869 10.1126/science.1141634 PMC2646098

[20] Scott LJ, Mohlke KL, Bonnycastle LL, Willer CJ, Li Y, et al (2007) A genome-wide association study of type 2 diabetes in Finns detects multiple susceptibility variants. Science 316: 1341–1345. 17463248 10.1126/science.1142382 PMC3214617

[21] Hertel JK, Johansson S, Sonestedt E, Jonsson A, Lie RT, et al (2011) FTO, type 2 diabetes, and weight gain throughout adult life: a meta-analysis of 41,504 subjects from the Scandinavian HUNT, MDC, and MPP studies. Diabetes 60: 1637–1644. 21398525 10.2337/db10-1340 PMC3292341

[22] Li H, Kilpelainen TO, Liu C, Zhu J, Liu Y, et al (2012) Association of genetic variation in FTO with risk of obesity and type 2 diabetes with data from 96,551 East and South Asians. Diabetologia 55: 981–995. 22109280 10.1007/s00125-011-2370-7 PMC3296006

[23] Barber TM, Bennett AJ, Groves CJ, Sovio U, Ruokonen A, et al (2008) Association of variants in the fat mass and obesity associated (FTO) gene with polycystic ovary syndrome. Diabetologia 51: 1153–1158. 18478198 10.1007/s00125-008-1028-6

[24] Ewens KG, Jones MR, Ankener W, Stewart DR, Urbanek M, et al (2011) FTO and MC4R gene variants are associated with obesity in polycystic ovary syndrome. PLoS One 6: e16390. 21283731 10.1371/journal.pone.0016390 PMC3024473

[25] Saxena R, Welt CK (2012) Polycystic ovary syndrome is not associated with genetic variants that mark risk of type 2 diabetes. Acta Diabetol. 10.1007/s00592-012-0383-4 PMC3679224 22389004

[26] Yan Q, Hong J, Gu W, Zhang Y, Liu Q, et al (2009) Association of the common rs9939609 variant of FTO gene with polycystic ovary syndrome in Chinese women. Endocrine 36: 377–382. 19859840 10.1007/s12020-009-9257-0

[27] Chen ZJ, Zhao H, He L, Shi Y, Qin Y, et al (2011) Genome-wide association study identifies susceptibility loci for polycystic ovary syndrome on chromosome 2p16.3, 2p21 and 9q33.3. Nat Genet 43: 55–59. 21151128 10.1038/ng.732

[28] Rotterdam ESHRE/ASRM-Sponsored PCOS consensus workshop group (2004) Revised 2003 consensus on diagnostic criteria and long-term health risks related to polycystic ovary syndrome (PCOS). Hum Reprod 19: 41–47. 14688154 10.1093/humrep/deh098

[29] Weisell RC (2002) Body mass index as an indicator of obesity. Asia Pac J Clin Nutr 11 Suppl 8S681–684.

[30] Purcell S, Cherny SS, Sham PC (2003) Genetic Power Calculator: design of linkage and association genetic mapping studies of complex traits. Bioinformatics 19: 149–150. 12499305 10.1093/bioinformatics/19.1.149

[31] Shi Y, Zhao H, Cao Y, Yang D, Li Z, et al (2012) Genome-wide association study identifies eight new risk loci for polycystic ovary syndrome. Nat Genet 44: 1020–1025. 22885925 10.1038/ng.2384

[32] Berulava T, Horsthemke B (2010) The obesity-associated SNPs in intron 1 of the FTO gene affect primary transcript levels. Eur J Hum Genet 18: 1054–1056. 20512162 10.1038/ejhg.2010.71 PMC2987405

[33] Grunnet LG, Nilsson E, Ling C, Hansen T, Pedersen O, et al (2009) Regulation and function of FTO mRNA expression in human skeletal muscle and subcutaneous adipose tissue. Diabetes 58: 2402–2408. 19587359 10.2337/db09-0205 PMC2750213

[34] Wahlen K, Sjolin E, Hoffstedt J (2008) The common rs9939609 gene variant of the fat mass- and obesity-associated gene FTO is related to fat cell lipolysis. J Lipid Res 49: 607–611. 18048838 10.1194/jlr.M700448-JLR200

[35] Velders FP, De Wit JE, Jansen PW, Jaddoe VW, Hofman A, et al (2012) FTO at rs9939609, food responsiveness, emotional control and symptoms of ADHD in preschool children. PLoS One 7: e49131. 23155456 10.1371/journal.pone.0049131 PMC3498333

[36] Kim JJ, Choi YM, Cho YM, Hong MA, Chae SJ, et al.. (2012) Polycystic ovary syndrome is not associated with polymorphisms of the TCF7L2, CDKAL1, HHEX, KCNJ11, FTO and SLC30A8 genes. Clin Endocrinol (Oxf). 10.1111/j.1365-2265.2012.04389.x 22443257

[37] Attaoua R, Ait El Mkadem S, Radian S, Fica S, Hanzu F, et al (2008) FTO gene associates to metabolic syndrome in women with polycystic ovary syndrome. Biochem Biophys Res Commun 373: 230–234. 18572014 10.1016/j.bbrc.2008.06.039

[38] Olszewski PK, Radomska KJ, Ghimire K, Klockars A, Ingman C, et al (2011) Fto immunoreactivity is widespread in the rodent brain and abundant in feeding-related sites, but the number of Fto-positive cells is not affected by changes in energy balance. Physiol Behav 103: 248–253. 21295049 10.1016/j.physbeh.2011.01.022

[39] Wu Q, Saunders RA, Szkudlarek-Mikho M, Serna Ide L, Chin KV (2010) The obesity-associated Fto gene is a transcriptional coactivator. Biochem Biophys Res Commun 401: 390–395. 20858458 10.1016/j.bbrc.2010.09.064 PMC2963669

[40] Gerken T, Girard CA, Tung YC, Webby CJ, Saudek V, et al (2007) The obesity-associated FTO gene encodes a 2-oxoglutarate-dependent nucleic acid demethylase. Science 318: 1469–1472. 17991826 10.1126/science.1151710 PMC2668859

[41] Qu F, Wang FF, Yin R, Ding GL, El-Prince M, et al (2012) A molecular mechanism underlying ovarian dysfunction of polycystic ovary syndrome: hyperandrogenism induces epigenetic alterations in the granulosa cells. J Mol Med (Berl) 90: 911–923. 22349439 10.1007/s00109-012-0881-4

[42] Tan S, Scherag A, Janssen OE, Hahn S, Lahner H, et al (2010) Large effects on body mass index and insulin resistance of fat mass and obesity associated gene (FTO) variants in patients with polycystic ovary syndrome (PCOS). BMC Med Genet 11: 12. 20092643 10.1186/1471-2350-11-12 PMC2824654

[43] Wojciechowski P, Lipowska A, Rys P, Ewens KG, Franks S, et al.. (2012) Impact of FTO genotypes on BMI and weight in polycystic ovary syndrome: a systematic review and meta-analysis. Diabetologia. 10.1007/s00125-012-2638-6 PMC3433670 22801903

[44] Wehr E, Schweighofer N, Moller R, Giuliani A, Pieber TR, et al (2010) Association of FTO gene with hyperandrogenemia and metabolic parameters in women with polycystic ovary syndrome. Metabolism 59: 575–580. 19913856 10.1016/j.metabol.2009.08.023

[45] Hatziagelaki E, Wagner R, Kantartzis K, Heni M, Linder K, et al.. (2012) Insulin Resistant Phenotype of Polycystic Ovary Syndrome does not Seem to be Caused by Variation in FTO. Horm Metab Res. 10.1055/s-0032-1321817 22847851

