# Supplementary material for: Common Variant rs9939609 in Gene FTO Confers Risk to Polycystic Ovary Syndrome
Source: PLoS One. 2013 Jul 1;8(7):e66250. doi: 10.1371/journal.pone.0066250 (PMC3698074; doi:10.1371/journal.pone.0066250)
Supplement: File S1. — (DOC) [file pone.0066250.s001.doc]

Title

Common variant rs9939609 in gene FTO confers risk to polycystic ovary syndrome

Running title

rs9939609 in FTO, susceptibility to PCOS

Authors' name and institutions:

Tao Li *^1,2^, Li You *^1,2,3,4^，Keliang Wu*^1,2,3,4^, Xiuye Xing^1,2^, Peng Wang ^1,2^, Linlin Cui ^1,2^, Hongbin Liu ^1,2,3,4^, Yuqian Cui ^5^, Yuehong Bian ^1,2,3,4^, Yunna Ning ^1,2,3,4^, Han Zhao ^1,2,3,4^, Rong Tang #^1,2^, Zi-Jiang Chen #^1,2,3,4^

*, the authors contributed equally to this paper

#, corresponding author

Institutions:

1. Center for Reproductive Medicine, Provincial Hospital Affiliated to Shandong University, Jinan, China

2. National Research Center for Assisted Reproductive Technology and Reproductive Genetics, Jinan, China

3. The Key laboratory for Reproductive Endocrinology of Ministry of Education, Jinan, China

4. Shandong Provincial Key Laboratory of Reproductive Medicine, Jinan, China

5. Department of Obstetrics and Gynecology, Renji Hospital, School of Medicine, Shanghai Jiaotong University, Shanghai, 200001, China

Corresponding author:

Zi-Jiang Chen

E-mail: chenzijiang@hotmail.com

Ground mail addresses: Shandong Provincial Key Laboratory of Reproductive Medicine, Center for Reproductive Medicine, Provincial Hospital Affiliated to Shandong University, 324 Jingwu Road, Jinan, 250021, China

Tel: 86-531-85187856

Fax: 86-531-87068226

Word count: 2248

Key terms: polycystic ovary syndrome, association study, fat mass and obesity-associated gene

**Index**

**Table S1** The detected SNPs in intron 1 of FTO from PCOS GWAS data

**Table S2** Clinical and metabolic data of 3 genotypes PCOS patients

**Table S3** Clinical and metabolic data of dominant model for PCOS patients

Table S1 The detected SNPs in intron 1 of FTO from PCOS GWAS data

|  |  |  | MAF | |  |  |
| --- | --- | --- | --- | --- | --- | --- |
| SNP | Minor allele | Major allele | PCOS | control | P | OR |
| rs13334933 | G | A | 0.2831 | 0.2864 | 0.8332 | 0.9837 |
| rs12446228 | A | G | 0.2681 | 0.2898 | 0.1699 | 0.8976 |
| rs9939973 | A | G | 0.1767 | 0.1502 | 0.04181 | 1.214 |
| rs9940646 | G | C | 0.176 | 0.1502 | 0.04721 | 1.208 |
| rs9940128 | A | G | 0.1767 | 0.1508 | 0.04651 | 1.209 |
| rs11075985 | A | C | 0.2074 | 0.1816 | 0.06428 | 1.179 |
| **rs9922047** | **C** | **G** | **0.324** | **0.3739** | **0.00305** | **0.8027** |
| **rs17817288** | **A** | **G** | **0.4045** | **0.4406** | **0.03837** | **0.8625** |
| rs1477196 | A | G | 0.266 | 0.2853 | 0.2214 | 0.9079 |
| **rs1121980** | **A** | **G** | **0.1774** | **0.1497** | **0.03308** | **1.225** |
| **rs7193144** | **C** | **T** | **0.1303** | **0.1031** | **0.01593** | **1.303** |
| rs16945088 | G | A | 0.07981 | 0.07567 | 0.6608 | 1.059 |
| **rs8057044** | **A** | **G** | **0.204** | **0.1766** | **0.04718** | **1.195** |
| **rs8050136** | **A** | **C** | **0.1241** | **0.1009** | **0.03604** | **1.263** |
| rs9936385 | C | T | 0.116 | 0.09753 | 0.08927 | 1.214 |
| rs11075989 | T | C | 0.1166 | 0.09753 | 0.07842 | 1.222 |
| rs11075990 | G | A | 0.1187 | 0.09753 | 0.05226 | 1.246 |
| ***rs9939609*** | ***A*** | ***T*** | ***0.131*** | ***0.09809*** | ***0.00247*** | ***1.23*** |
| rs7201850 | T | C | 0.1623 | 0.1446 | 0.162 | 1.146 |
| rs7185735 | G | A | 0.1173 | 0.09978 | 0.1083 | 1.199 |
| rs9931164 | G | A | 0.05116 | 0.04765 | 0.645 | 1.078 |
| **rs9941349** | **T** | **C** | **0.1767** | **0.1491** | **0.03359** | **1.225** |
| rs7190492 | A | G | 0.01023 | 0.01233 | 0.5745 | 0.828 |
| **rs9930501** | **G** | **A** | **0.1869** | **0.1536** | **0.0116** | **1.267** |
| **rs9930506** | **G** | **A** | **0.1883** | **0.1547** | **0.01124** | **1.267** |
| rs6499646 | C | T | 0.4666 | 0.4524 | 0.4182 | 1.059 |
| rs17218700 | A | G | 0.08322 | 0.102 | 0.06705 | 0.799 |

MAF=minor allele frequency. SNPs with P＜0.05 have been expressed in bold type.

Table S2 clinical and metabolic data of 3 genotypes PCOS patients

|  | Genotype AA | Genotype AT | Genotype TT |
| --- | --- | --- | --- |
| AGE | 28.82±4.19 | 28.16±3.65 | 28.40±3.77 |
| **BMI ^a,b,c^** | **26.99±4.44** | **25.30±4.43** | **24.60±4.21** |
| **WAIST** **^a,b,c^** | **90.53±12.69** | **85.14±11.87** | **83.89±11.33** |
| WHR | 0.87±0.07 | 0.86±0.06 | 0.86±0.06 |
| FSH | 6.29±1.60 | 6.27±1.66 | 6.28±1.77 |
| LH | 11.10±5.40 | 10.74±6.79 | 10.58±7.05 |
| LH/FSH | 1.82±0.93 | 1.77±1.07 | 1.74±1.12 |
| T | 58.65±27.55 | 54.54±25.18 | 55.67±24.45 |

The data were presented as mean ± SD. WHR=ratio of waist to hip. FSH=follicle stimulating hormone. LH=luteinizing hormone. T=testosterone. A=the comparison of genotype AA vs. genotype AT is statistically different (P<0.05). b= the comparison of genotype AT vs. genotype TT is statistically different(P<0.05). c=the comparison of genotype TT vs. genotype AT is statistically different(P<0.05).

Table S3 clinical and metabolic data of dominant model for PCOS patients

|  | Genotype AA+AT | Genotype TT | P | P adj |
| --- | --- | --- | --- | --- |
| AGE | 28.20+3.70 | 28.40+3.77 | 0.17 | - |
| BMI | 25.42+4.45 | 24.60+4.21 | 6.24E-07 | - |
| GLU-0` | 5.25+0.79 | 5.30+0.95 | 0.25 | 0.154 |
| GLU-30` | 8.45+1.79 | 8.54+1.87 | 0.38 | 0.880 |
| GLU-60` | 8.54+5.01 | 8.34+2.84 | 0.29 | 0.394 |
| GLU-120` | 6.67+3.50 | 6.62+2.24 | 0.64 | 0.511 |
| GLU-180 | 4.82+1.41 | 4.99+1.63 | 0.04 | 0.467 |
| INS-0` | 12.76+9.87 | 12.29+8.22 | 0.18 | 0.994 |
| INS-30` | 75.12+48.25 | 75.18+50.75 | 0.98 | 0.904 |
| INS-60` | 88.74+60.49 | 88.05+65.14 | 0.84 | 0.783 |
| INS-120` | 70.97+67.05 | 68.93+60.69 | 0.44 | 0.293 |
| INS-180 | 25.42+33.20 | 26.03+33.46 | 0.73 | 0.846 |
| HOMA-IR | 3.11+3.23 | 2.97+2.33 | 0.21 | 0.954 |
| CHOL | 4.53+0.93 | 4.55+0.94 | 0.62 | 0.302 |
| TG | 1.30+0.86 | 1.31+0.96 | 0.74 | 0.206 |
| HDL-C | 1.30+0.37 | 1.33+0.58 | 0.38 | 0.701 |
| LDL-C | 2.78+0.93 | 2.78+0.93 | 0.90 | 0.259 |

P adj=adjusted P value by BMI in logistic regression. Dominant model: Genotype AA+AT vs. Genotype TT. GLU=glucose. INS=insulin. HOMA-IR=homeostasis model for insulin resistance. CHOL=cholesterol. TG=triglycerides. HDL=high density lipoprotein. LDL=low density lipoprotein.
